# What makes a mountain?

**DOI:** 10.1007/s13280-026-02356-4

**Published:** 2026-03-13

**Authors:** Øyvind Paasche, Helle Siljeholm

**Affiliations:** 1 Department of Climate Dynamics, NORCE, Bergen, Norway; 2 Bjerknes Centre for Climate Research, Bergen, Norway; 3 Oslo National Academy of Arts, Oslo, Norway

**Keywords:** Art, Mountain, Nature, Preservation, Restoration, Value

## Abstract

Mountains are visually striking landforms that influence climate, ecosystems, and biodiversity through the steep gradients they create. Their dynamic environments attract diverse life forms, including humans—who depend on mountains for resources, refuge, and inspiration. Human concern with protecting and managing these landscapes has led to legal designations such as national parks, nature reserves, and, increasingly, restoration initiatives. Yet efforts to define mountains universally remain contested: global mapping suggests they cover between 12 and 30% of Earth’s land surface, but mountains beneath ice sheets or oceans remain excluded. To explore how perceptions of mountains shape their definition and value, we examine three European cases: Mt. Snøhetta (Norway), the Dolomites (Italy), and Mt. Parnassos (Greece). Each illustrates how science, myth, and culture inform human–mountain relationships. We introduce the “Næss dilemma,” highlighting the paradox that while empathy for mountains depends on interaction, such engagement inevitably alters what we seek to preserve.

## Introduction

A mountain generates movement by not moving. Air masses are forced to rise over it, releasing rain and snow in the process. Pressure and temperature gradients set winds in motion. As stable airflows approach a mountain, they often divert around it, accelerating through passes and down steep slopes in turbulent formations known as lee wave trains (Barry 2008).

Perennial snowfields high on the mountain feed brooks and streams through the summer months, flowing as long as snow and temperature conditions allow (Bormann et al. 2018). Rockfalls, landslides, debris flows, and avalanches cascade down the steeper slopes, settling on scree fields, glaciers, and valley floors (Fischer et al. 2012). Movement of air, water, or ice takes place around and across the mountain without the mountain itself moving. By holding its position, it becomes a magnet for life. Animals, plants, trees, and birds are drawn to the mountain, where they establish diverse ecosystems. The altitudinal gradients create shifting envelopes of opportunity, fostering adaptation, speciation, and biodiversity (Perrigo et al. 2019). As elevation increases, summers grow shorter and winters longer enabling a wide seasonality span.

Mountains attract many forms of life, including humans. Given our genetic adaptations to high-altitude living (Moore 2016), it seems likely that humans have been drawn to mountains since our African origins some 2.8 million years ago (Villmoare et al. 2015). We are likewise forced to navigate the challenges mountains pose by crossing them, settling among them, and using them for shelter and refuge. Human settlement in mountainous regions has occurred both temporarily and permanently, and some scholars suggest that the rise of mountain ranges over the last 4 million years may have influenced human evolution itself (Molnar 1990).

This fundamental asymmetry—between the immobility of the mountain and the mobility of everything around it—generates movement, fosters relationships, and shapes perceptions (Peattie 1936; Price 1981; Gerrard 1990; Messerli and Ives 1997). Perhaps it was this quality, so present in the Greek landscape, that inspired Aristotle’s concept of a mover that is not itself moved. In Metaphysics, he writes: “…since what is moved and moves something is something medial, there is something that moves without being moved, being eternal, substance, and activity. This, though, is the way the object of desire and the intelligible object move things: they move them without being moved” (Cohen and Reeve 2025).

Mountains are not immune to change. Aside from tectonic movement, they are increasingly affected by climate change and human activity (Pepin et al. 2022; Adler et al. 2022). Permafrost that has accumulated for millennia is thawing rapidly (Smith et al. 2022). These invisible internal shifts often manifest externally, destabilizing slopes and triggering rockfalls and avalanches. The retreat of permafrost and snowfields brings new vegetation patterns in their wake (Choler et al. 2024). Meanwhile, humans continue to build in and through mountains by constructing roads, tunnels, towns, and mines.

Landscapes, of which those dominated by mountains are but one, refuse to be disciplined because their roots reach into different strata of history, culture, and time (Bender 2002). According to Barabara Bender, academics have been slow to realize this notion, which may be true, but it does little to quench the public need for definitions.

To explore these questions, we examine three specific European cases: Mt. Snøhetta in Norway, the Dolomites in Italy, and Mt. Parnassos in Greece. Each represents a different way of seeing and relating to mountains be that through science, history, art, or myth. While these cases are geographically limited, they illustrate tensions in how mountains are defined and valued that resonate beyond Europe.

We also introduce what we term the “Næss dilemma,” which frames the paradox of environmental empathy: we cannot develop a relationship with nature and mountains without engaging with it, but that very engagement risks altering or degrading the object of our attention. In short, this study explores how mountains are defined, how we interact with them across time, and how these interactions shape our perceptions which in effect guide how we interpret, protect, exploit, or restore them.

## How to define a mountain?

### Why definitions matter

The definition of a landform reflects not only how well we understand it but also its operability—how it functions within scientific, political, and cultural systems—and the degree to which consensus can be reached (e.g., Price et al. 2019). In many cases, concepts like “mountain” existed long before scientific definitions were attempted. As a result, definitions may conflict with societal or cultural understandings. A mountain in a relatively flat landscape may be perceived very differently than one in a more rugged setting.

The need for functional, precise definitions arises from various motivations: scientific classification, cultural identity, legal jurisdiction, and environmental policy (Price et al. 2019). Technological progress also enables new approaches, which in turn can lead to the revision or refinement of definitions which is certainly true for mountains. The International Panel of Climate Change’s Sixth Assessment Report (IPCC–AR6) offers one such definition: “A mountain is a landform formed through plate tectonics that rises above its surrounding area, characterized by verticality and ruggedness such as gentle or steep sloping sides, sharp or rounded ridges, and a high point called a peak or summit” (Adler et al. 2022).

This authoritative definition combines both relative and absolute terms. The phrase “rises above its surroundings” is relational and also applies to hills, tablelands, and eroded mountain ranges. The core constraint lies in the phrase “formed through plate tectonics,” which ties mountain identity to a specific geodynamic process. Note that this framing risk excluding other formative processes. Glaciers and climate play substantial roles in shaping mountains (Molnar and England 1990; Willett 1999). If plate tectonics defines the origin of a mountain, processes like glacial erosion may be relegated to a merely modifying role. This challenges concepts like the “glacial buzzsaw” (Mitchell and Montgomery 2006), which positions glaciers as mountain-makers in their own right.

**Fig. 1:**
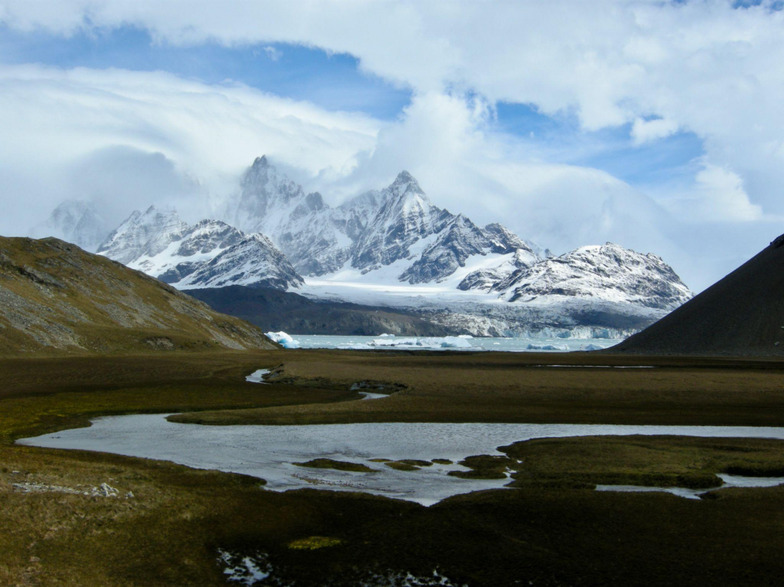
Jagged alpine mountains in Cumberland West Bay on the remote island of South Georgia (54° S, 36° W), located in the sub-Antarctic zone. The mountains, known as the “Three Brothers,” rise from approximately 1500 m in elevation down to sea level, where drifting icebergs float by as observed by the author in 2008. On the island, both cirque and tidewater glaciers are undergoing an unprecedented retreat in the context of the past 14 000 years (Bakke et al. 2021), suggesting that they, like so many other mountain landscapes, are in a state of transition. Photo: Øyvind Paasche

Ice sheets, glaciers, and rivers not only carve landscapes but also deposit sediment, reshaping relief and ruggedness. Heavily incised valleys may later be filled with glacial deposits, significantly altering the topography. As these landscapes deglaciate, their elevation, slope, and texture change—complicating efforts to define mountains solely by verticality or ruggedness. Limitations apart, the IPCC definition is useful and widely applicable (Fig. 1).

### A quantitative approach

Despite some reluctance within the scientific community to formally define mountains (e.g., Gerrard 1990), efforts toward quantitative classification have steadily progressed. Kapos et al. (2000) laid the groundwork for one such approach, estimating that 24% of the global land area or about 35.8 million km^2^ could be classified as mountainous. This would make mountains among the most extensive landforms on Earth (Cf. Körner et al. 2011; Adler et al. 2022) although figures range between 12.5 and 30%, depending on criteria (Sayre et al. 2018; Körner et al. 2021), and regional estimates vary even more. In Norway, for instance, mountain coverage has been estimated to range from 30 to 93% of the national territory, depending on whether mountains are assumed to begin at 300 m or extend to sea level (Price et al. 2019).

### The importance of unaccounted surfaces

The wide variance in mountain coverage reflects differences in theory, methodology, and intended application. A major omission in most estimates is Antarctica covering 14 million km^2^, 98% of which is covered by ice. Beneath the Antarctic Ice Sheet (AIS), entire mountain ranges such as the Gamburtsev Mountains remain preserved. Formed 14–15 million years ago (Sugden and Jamieson 2018), these ranges have been shielded from erosion and remain remarkably intact. A similar situation is partly true for the Greenland Ice Sheet as well (Paxman et al. 2024), although at a smaller scale.

If subglacial mountains were included in global estimates, the total area classified as mountainous would increase substantially. Another major exclusion in most models is the ocean floor. The oceanic lithosphere is teeming with mountains—some of the most prominent being the mid-ocean ridges, which span over 65 000 km, far surpassing any terrestrial range.

Seamounts are predominantly volcanic peaks rising up from the ocean floor but not breaking the sea surface. Once they breach the surface, they would be categorized as islands. Subtract the ocean, and these submerged peaks would be recognizable as mountains. The altitude of seamounts was initially set to 1000 m (Menard 1964) but has later been lowered. Kim and Wessel (2011) mapped 14 500 seamounts, with around 8500 exceeding 1000 m in height. An updated mapping by Gevorgian et al. (2023) places the number of seamounts to 43 454, although they include observations below 1000 m, but the total number may be much higher (Wessel et al. 2010). Harris et al. (2014), using a geomorphological lens, defined "abyssal mountains" as features with a minimum relief of 1000 m. They estimate 17.7% of the ocean floor which is a reminder that mountain geographies extend well beyond the terrestrial part of the planetary surface.

### Alternative approaches to mountain definitions

Every mountain definition draws a boundary, and boundaries have consequences—legal, ecological, cultural, and economic (cf. Cosgrove 1985). Often, the impetus for a definition is less about scientific clarity than political or bureaucratic necessity. The result is usually a pragmatic compromise: a quantitative formula tailored to data availability, resolution, and policy needs but one that may say little about how people experience mountains. In a survey conducted by Smith and Mark (2001), respondents were asked to name known geographic features, and “Mountains” topped the list. This suggests that mountains hold a strong place in the human imagination, irrespective of measurable characteristics.

**Fig. 2:**
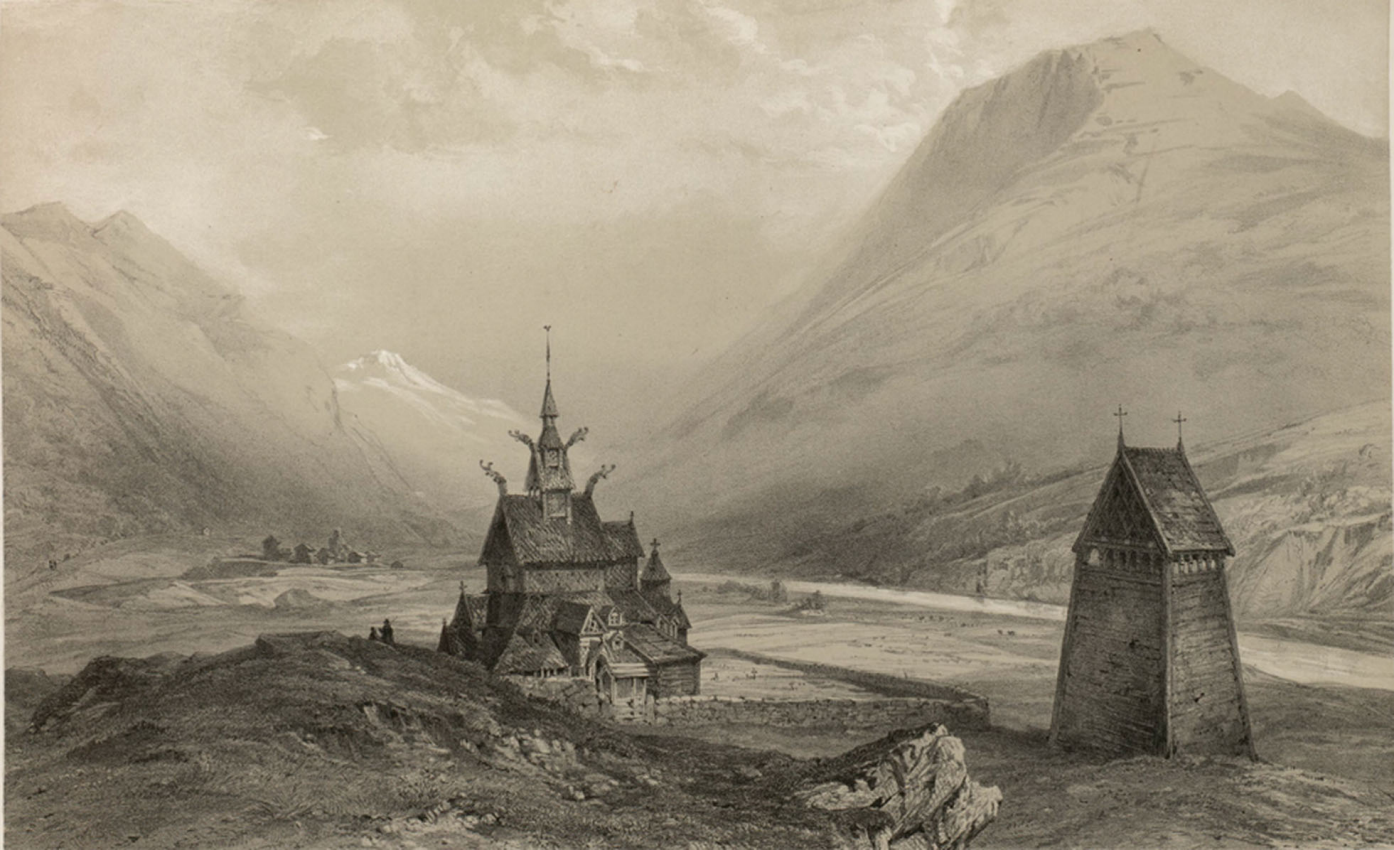
Borgund stave church, centrally located in a valley in western Norway, with the river Lærdalselvi flowing discreetly in the background. A freestanding bell tower stands to its right. The stave church has been dated by dendrochronology to 1180 CE. The image, titled “Vallée et Église de Borgund” and catalogued as #42, was drawn by Auguste Étienne François Mayer (1805–1880), who participated in the French scientific expedition led by Charles Joseph Barthélémy Giraud (1802–1881)

Most definitions draw on the three core parameters: elevation, relief or ruggedness, and slope angle. In everyday language, this might translate to “something high, steep, and uneven.” Yet these terms exclude the experience of the mountain including the emotional and aesthetic impact of encountering one. Arne Næss, philosopher and mountaineer, called this “the mountain’s greatness”: its capacity to evoke awe and deep connection (Fig. 2).

Throughout history, mountains have been linked to the spiritual and the divine (see Bernbaum 2022). In Norway, stave churches built nearly 900 years ago can be said to resemble the mountain forms around them with their steep roofs, narrowing spires, and tiered layers mimicking the peaks nearby. One could imagine the mountain as the model for the church, not just its backdrop but enabling the sensation of the mountain embodied in a church.

In Investigative Aesthetics: Conflicts and Commons in the Politics of Truth, Fuller and Weizman (2021) describe sense-making as a “poly-perspective assemblage,” akin to cubist painting; a convergence of perspectives that opens pathways for contestation and new interpretations. This method of “sensing” can be useful in understanding mountains as cultural artifacts, more-than-physical entities and material witnesses (Schuppli 2020). In a political and cultural context, mountains do not only symbolize purity and grandeur; they also bear the scars of destruction: mountaintop removal (Boyles et al. 2017), nuclear subsidence (Wang et al. 2018), and the cultural erasure caused by war.

## Three considerations on mountains

**Fig. 3:**
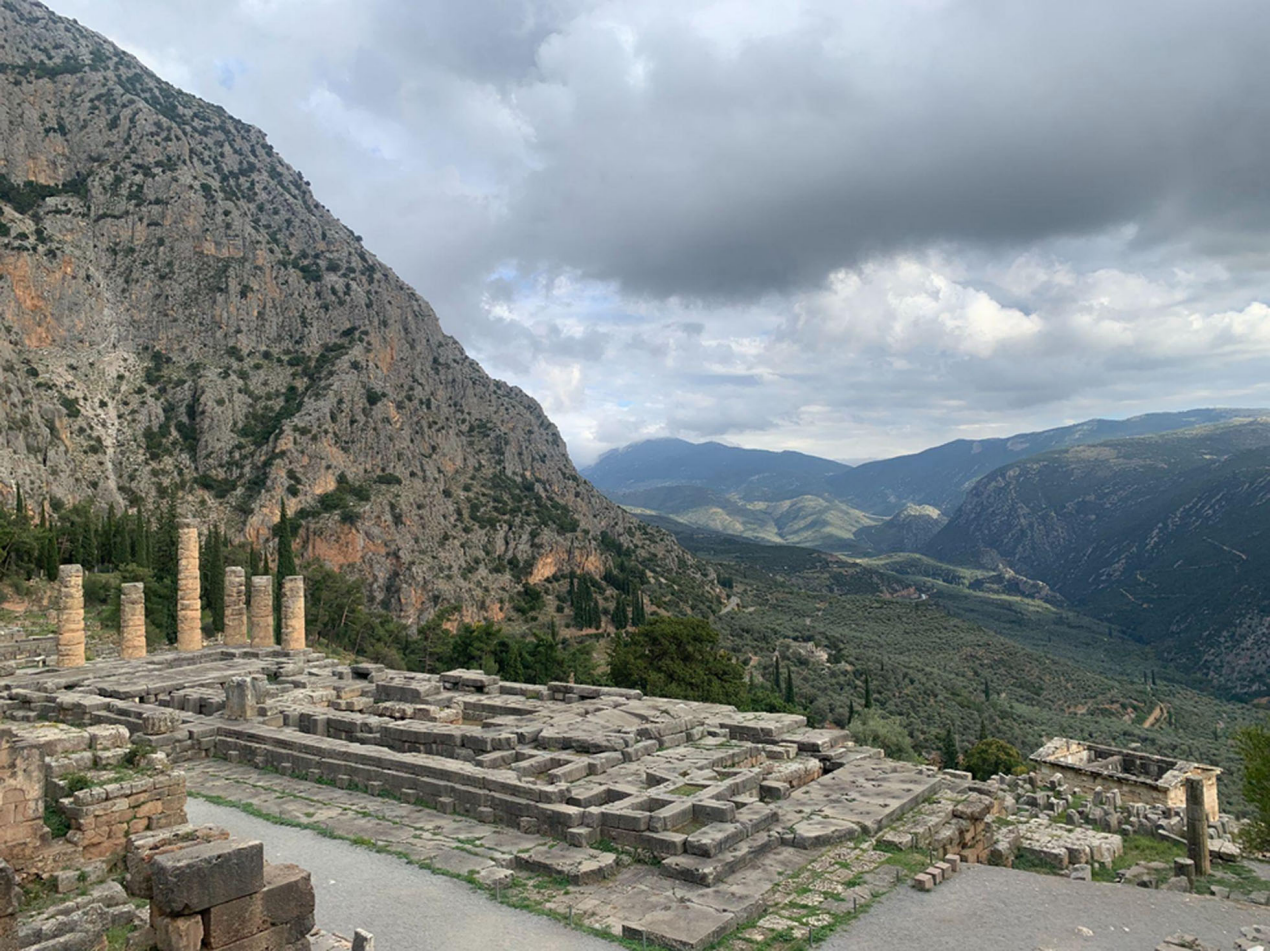
Visible ruins of the Temple of Apollo at Delphi, located on Mount Parnassos in central Greece. In antiquity, the temple was home to the Oracle of Delphi, the high priestess Pythia. The Oracle recruited local women over the age of 50, who sat upon a designated tripod inside the sanctuary, holding laurel leaves and inhaling fumes that rose from cracks in the rock beneath the temple—a ritual performed prior to delivering prophetic messages. Delphi was already a major Pan-Hellenic religious site by the eighth century BCE. Several iterations of the temple have been built at this site; the ruins that remain today date from the fourth century BCE. Photo: Helle Siljeholm

Our starting point is that many societies have their own iconic mountains or places of historical meaning which, in effect, shape those societies’ perception of what a mountain is, what it contains, and what its social and political significance might be. At the same time, some cultures may not regard mountains positively, underscoring the diversity of human–mountain relationships (Fig. 3).

### The mythological mountain: Mt. Parnassos and its oracles

Mt. Parnassos, reaching an altitude of 2457 m, lies in a mountain range in central Greece. The mountain is central to one of the most celebrated myths of early Greek culture; myths that have had a significant impact on Western civilization and beyond (Scott 2014). On the western side of the range lies the Pan-Hellenic Sanctuary of Delphi, a place hailed by the ancient Greeks as the center of the universe. The sacred symbol of this belief was a carved oval stone called the “Omphalos,” meaning “navel of the world” (Scott 2014).

Apollo, the god of divination, archery, sports, and music, ordered a temple to be built in his honor after slaying Python, the monstrous serpent that guarded the oracles of the site for its original custodian, Gaea being the goddess of Earth. According to Homer’s Hymn to Apollo, Apollo left Python to rot in the sun. The decaying body inspired the name of the Oracle that succeeded Gaia’s: Pythia. Her name is derived from pythin (to rot) and the site’s Bronze Age name, Pytho (Hayward 2020).

From the seventh century BCE until the late fourth century CE, Pythia—the high priestess, selected from among local women in their fifties—was one of the most powerful women in the classical world. Eyewitness accounts describe her seated on a tripod in a separate chamber of the sanctuary, inhaling vapors rising from chasms in the earth. These vapors induced a trance-like state from which she delivered prophecies—some of which still resonate today.

Pythia was said to be the most precise oracle of her time (Crosby 2022). Her audience (those who could afford it) traveled from near and far to consult her, typically on matters of war and power. Between 535 and 615 of Pythia’s prophecies are preserved today as records for future generations. The phrase “Know thyself” is famously carved into a stone column outside the sanctuary.

In 1892, a team of French archeologists, working with German and Greek colleagues, embarked on La Grande Fouille (“The Great Excavation”) at Delphi. They made many important discoveries but, surprisingly, did not uncover the expected reservoir of natural gas beneath the sanctuary (Broad 2007). This baffled the archeologists. Could the entire tradition have been a theatrical performance repeated over a thousand years by a group of euphoric middle-aged women? After all, it was said that hardly anyone could fully understand Pythia’s utterances, and that she herself could never recall her prophecies after the trance ended.

The image of a female oracle inhaling fumes from the inner earth and channelling the future proved too wild for the rational minds of the modern age. Yet scepticism about her powers predates even that era. In the mid-1990s, a group of geologists returned to Mt. Parnassos to re-examine the Delphi site (De Boer and Hale 2000a; b). They began to realize that the classical-era witnesses might have been right. Vapors had likely escaped from rocks beneath Apollo’s Adyton (inner sanctuary), but geological shifts had displaced much of the evidence.

These vapors, caused by seismic ruptures in the earth, were likely hydrocarbon gases (De Boer and Hale 2000a; b). When inhaled, such gases can induce symptoms that align with descriptions of Pythia’s trance: euphoria, slurred speech, memory loss, and altered perception (Broad 2007). A photograph from The Great Excavation even shows traces of vapor, accompanied by a note referencing the finding, though this detail was apparently lost in translation in later reports (Broad 2007).

A more recent study by Piccardi et al. (2008) disputes the type, source, and role of the gas, offering an alternative explanation. They suggest that hydrogen sulfide (H_2_S), a gas often released by earthquakes and known for its rotten-egg smell, may have been the original myth’s trigger. This theory predates Apollo’s story and links back to Gaea, the first ruler of the oracle cult. Referring again to the Hymn to Apollo, the vivid descriptions of Python’s death—visual, auditory, and olfactory—could be interpreted as symbolic of an earthquake. Indeed, a common feature across ancient Greece was to build sacred sites atop seismic zones (Piccardi, pers. comm.).

The geologists propose a metaphysical link between the earth’s movements (the mountain) and cultural expression. Pythia and Python can thus be understood as symbolic revivals of the mountain, animated by the subterranean breath of Mother Earth. Moving beyond anthropocentric interpretations of the gods, we return to the ever-present spirits of nature whose messages remain unresolved in the myths. What, then, do the bouldering rocks prophesy? We are left to wonder.

### The transformative mountain: The dolomites

**Fig. 4:**
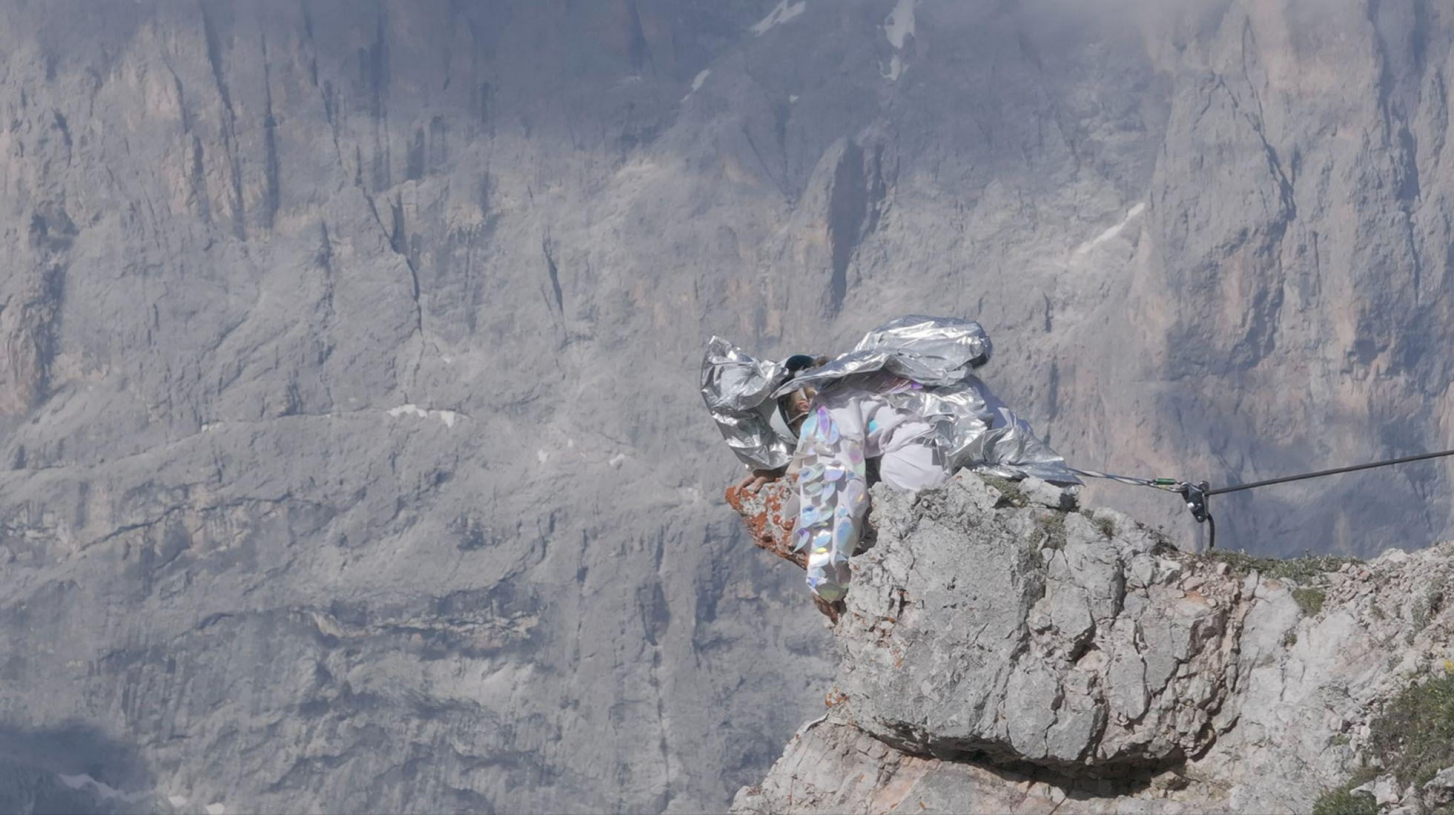
Video still by Fred Arne Wergeland from The Mountain Body, Sea of Rocks by the artist Helle Siljeholm (2024). The Mountain Body, Sea of Rocks is a choreographic performance for five climbers and performers, staged across four cliff faces in the Dantercepies mountain formation in the Dolomites. The piece premiered on July 13, 2024, at the Bolzano Danza/Tanzbozen Festival and was co-presented by Biennale Gherdëina 9—The Parliament of Marmots. The image shows performer Pernille Holden positioned on one of the four peaks in the Dantercepies formation. The mountain wall in the background is part of the atoll-like reef known as Langkofel–Sassolungo. The costume, designed by Karine Faou, draws inspiration from fossils of marine creatures found in the geological archive of the Dolomites

The limestone plateaus of Delphi offer stunning views toward the Corinth Sea, once part of the ancient Tethys Ocean, named after the Greek Titan goddess, daughter of Gaea. If one follows the ancient seabed northward to Venice, Italy, and then along the Piave River, a vast landscape unfolds: fifteen immense mountain formations that today make up the Dolomites (Bosellini et al. 2003) (Fig. 4).

In June 2009, the Dolomites were inscribed on the UNESCO World Heritage List, recognized for multiple criteria including Criterion VII, which highlights their extraordinary aesthetic and landscape value. According to the official statement: “The Dolomites are widely regarded as being among the most attractive mountain landscapes in the world. Their intrinsic beauty derives from a variety of spectacular vertical forms such as pinnacles, spires and towers, with contrasting horizontal surfaces including ledges, crags and plateaux, all of which rise abruptly above extensive talus deposits and more gentle foothills […]Some of the rock cliffs here rise more than 1500 m and are among the highest limestone walls found anywhere in the world.” (Dolomiti UNESCO Foundation).

Lying down in the midst of this lush, flowering alpine landscape—at approximately 3500 m—one might suddenly realize that this very spot, at the summit of the Alps, was once a coral bed at the bottom of the ancient Tethys Ocean, some 250 million years ago (Gianolla and Andreetta 2008). The ancestors of today’s birds circling overhead were not hunting marmots and mountain mice but diving for mussels and fish in a bustling tropical sea. What sounds once filled this landscape we can only imagine but perhaps a soundscape ranging from low, droning vibrations to high-pitched whistles. A polyphonic sea rave composed of corals, algae, sponges, mussels, and perhaps even ichthyosaurs, set amid vivid volcanic activity. Sound travels faster in water than in air, allowing marine animals to communicate across vast distances (Simmonds et al. 2013). In the ocean, sound waves ignore human-made boundaries, transcending borders and bypassing resource claims and extractive activity. Across the vast underwater mountain ridges, many of which remain unrecognized, the sound continues.

The Dolomites are formed from a rare composition of marine sediment, coral, and algae-based dolomite rock (CaMg(CO_3_)_2_) (McKenzie et al. 2009). In many areas, the mountains are etched with diagonal striations that span entire valleys including natural notations of the African continental plate’s subduction beneath the Eurasian plate. This monumental collision lifted the ancient coral reefs from the ocean floor, folding and thrusting them upward to form the Alpine peaks. Arne Næss described “self-realization” as the moment when the small self—the human being—recognizes itself as part of the larger self: nature (Næss 1989). Lying in the vast alpine terrain, contemplating the transformation from seabed to summit becomes an act of ecological meditation—a reflection on the deep entanglement of human and non-human bodies within interconnected ecosystems.

This ongoing tectonic activity continues to shape the landscape today. The Alps are still rising with rates of 1 mm/year in the east and central regions, and up to 2.5 mm/year in the west (Sternai et al. 2019). These vertical displacements are partly the result of deglaciation and erosion following the last Ice Age. However, human-induced climate change and direct interventions may now accelerate erosion and intensify climate-related hazards—thereby altering our perception of mountains once celebrated for their natural beauty.

In January 2024, the Norwegian government provoked widespread concern when it announced plans to open selected areas of the seabed for mineral prospecting. The decision is controversial for many reasons, including insufficient understanding of seabed ecosystems, rising noise and light pollution in deep-sea environments, and criticism from environmental scientists. Some even called for Norway to resign its position as Co-Chair of the High Level Panel for a Sustainable Ocean Economy (Amon et al. 2024; Crane et al. 2024). Seabed mountains and submerged landforms often lack visibility, recognition, and protection making them especially vulnerable to extraction and disruption (Rogers 2018). This raises a pressing question: how can societies protect and value landscapes they cannot see?

It is not only humans but also plants, animals, and technologies that function as sensing bodies some of which, as Fuller and Weizman (2021) argue, possess sense-making capacities. By including these “more-than-human” actors in the assemblage of the commons, they propose an expanded concept of aesthetics—one that exceeds mere visual perception and engages directly with political, ecological, and legal realities. “Aesthetics is not a question of beautification but of the sensible,” Weizman notes in the interview “Why Aesthetics Must Mean More Than Beauty” (O’Mahony 2022).

In the case of the Dolomites, a UNESCO World Heritage landscape, the mountains have yet to see their submerged counterparts, formed in the same Tethyan oceanic system, recognized in a similar way. Though part of the same geomorphological lineage, these underwater mountain bodies remain unnamed, unmapped, and unprotected. Could an expanded notion of aesthetics help us make sense of nature both above and below the ocean’s surface? Could it provide a path toward recognizing the mountain body in its totality and not just the limbs that rise above the sea, but also those still hidden beneath it? Perhaps it is time to enlarge the family of mountain definitions.

### The modern mountain: Dovre and Snøhetta of Norway

**Fig. 5:**
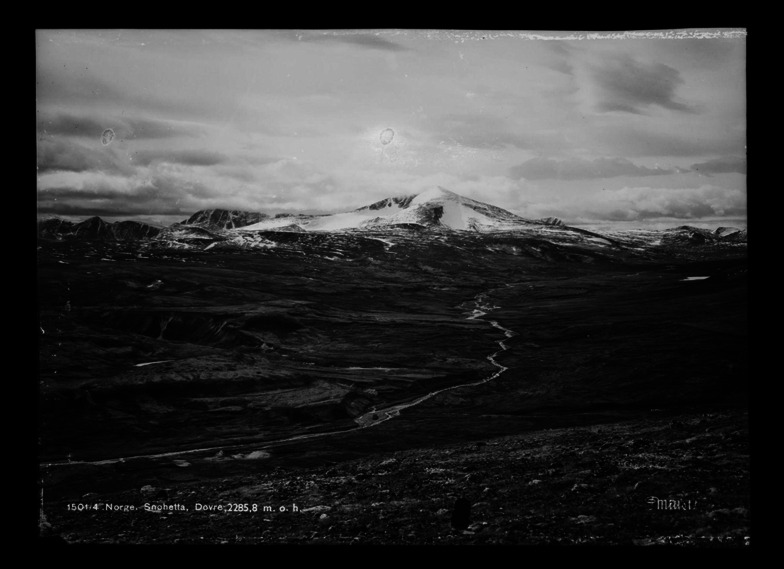
The iconic Mt. Snøhetta (which translates to “the snowhood”), viewed from the east, shows its snowy summit and two of four cirques—three of which still contain glaciers. During the last deglaciation, the mountain formed a nunatak (Lane et al. 2020). From its summit, glaciers retreated down adjacent valleys, leaving large sedimentary deposits that were later eroded by meltwater. Terraces, evidence of these processes, can be seen here at the mouth of the Stroplesjøen Valley, visible in the left foreground of the image. This historic photograph, taken in 1945, is one of many captured of the mountain since at least the 1890s. Photo credit: Mittet og Co (photographer unknown)

**Fig. 6:**
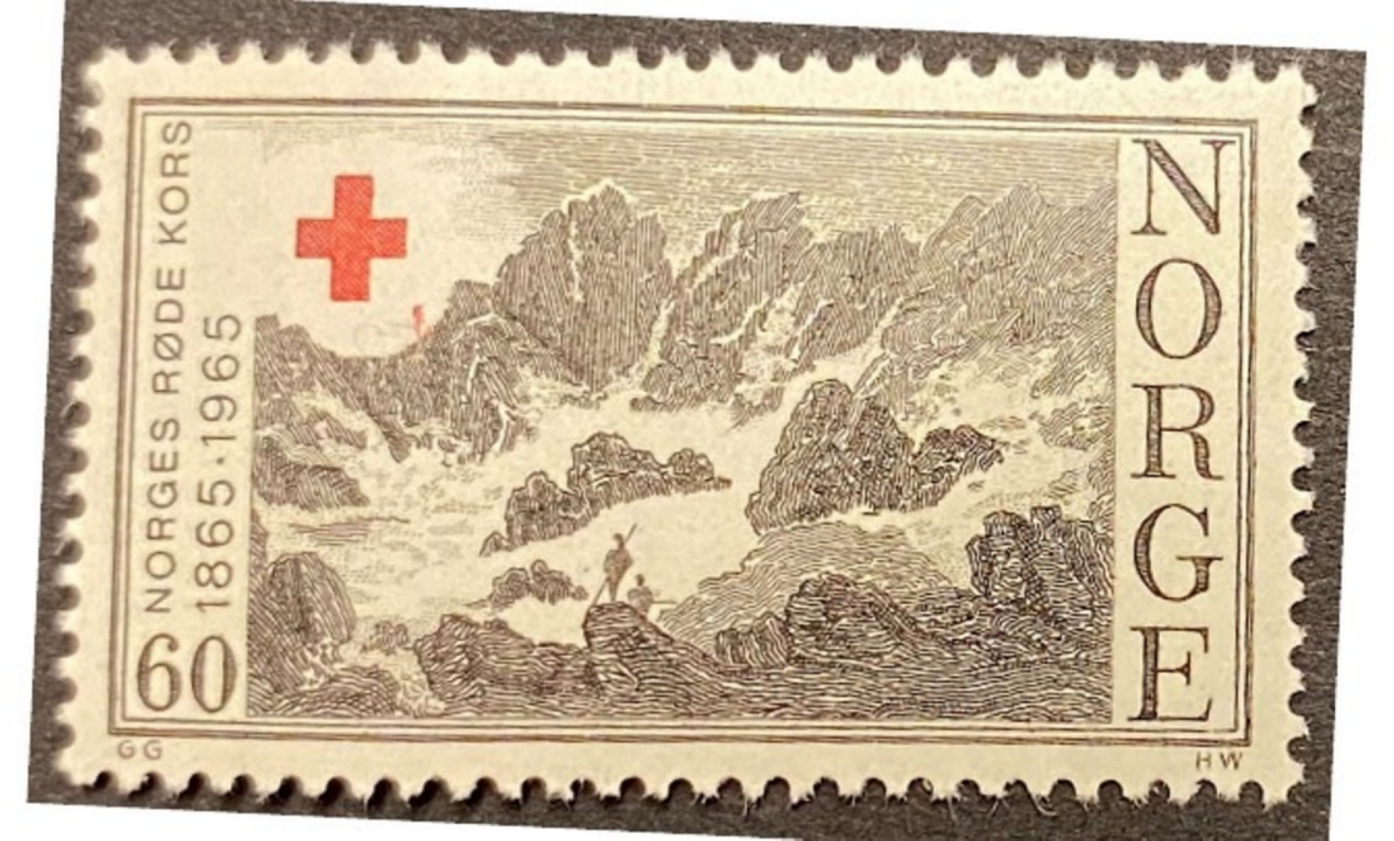
Drawing by Sir Thomas Dyke Acland (1787–1871), published in Alex Lamotte’s Voyage dans le Nord de l'Europe, consistant principalement de promenades en Norwege … dans l'année MDCCCVII (1813). The original drawing was reinterpreted for a commemorative national stamp series produced in collaboration with the Red Cross. The interplay between humans and nature is made explicit by the two explorers positioned on a ridge overlooking the glacier. They are set against the white expanse of the glacier surface, creating the contrast needed to distinguish their figures from the surrounding wild and dramatic landscape

The nature that shapes our perception is the nature we engage with (Fig. 5). Longstanding transport routes reinforce this engagement, leaving repeated impressions which is evident in countless images, photographs, and even a reproduction of a 19th-century English expedition on a national stamp (Fig. 6). The passage past Dovre and Mt. Snøhetta (2286 m), in central Norway, has been a preferred route for more than a thousand years. This north–south corridor, which runs alongside Mt. Snøhetta, is deeply woven into Norwegian history, myth, and culture to the extent that the mountain itself has become a national reference landform. When pledging allegiance to the Norwegian Constitution of 1814, its drafters swore they were “united in agreement until the mountains of Dovre [synonymous with Mt. Snøhetta] come apart.”

The choice of this mountain range as a symbol was not arbitrary. According to legend, King Harald Hårfagre (Fairhair), at just five years old, rescued a captive troll from certain death. As a result, he became an outcast, eventually taken in and raised by the same troll he had freed. The troll taught him the ways of the world, and years later, Harald returned not only to claim the throne, but to become the first king to unify Norway (Koht 1955). He ruled for 60 years, from 870 to 930 CE. The saga of King Fairhair, recorded in the medieval manuscript Flatøyboka (Codex Flatöiensis), is rich in symbolism. Perhaps its most enduring lesson is that one cannot govern a country like Norway without understanding nature—especially the mountains—which offer a unique lens on both human and natural realms.

“In modern times, Dovre is neither the home of trolls nor the symbol of the nation, but instead a place to enjoy nature,” wrote Professor Francis Bull in 1923 (Bull 1923). At that time, nature appreciation in a modern sense was largely limited to the well-off, but access was rapidly expanding. With the completion of the north–south railway in 1921, central Norway’s mountains became far more accessible, particularly for urban dwellers. Alongside this development came increased human activity, including the establishment of a 165 km^2^ military firing range in 1921. The site remained in active use until 2008. In 1969, mining operations began at Tverrfjellet near Hjerkinn, adjacent to Mt. Snøhetta. Over 25 years, the mine extracted nearly a ton of gold, 99 tons of silver, and 15 million tons of ore, reaching depths of 690 m (Dovre Municipality 2019).

The closure of the firing range in 2006 and the mine in 2014 marked the beginning of Norway’s most ambitious nature restoration initiative to date. Spearheaded by the Norwegian Defence Estate Agency and carried out in close collaboration with researchers and private contractors, the project aimed to rehabilitate a landscape considered a national reference. This included removing roads, clearing ammunition and explosives, and replanting vegetation to restore ecological function. As noted by Hagen et al. (2022), Norway remains in its early stages of large-scale ecological restoration. Still, the Dovre project reflects a significant shift in how mountain value is understood, not only in terms of ecosystem services like carbon uptake and biodiversity (Martín-López et al. 2019), but also in how such landscapes are culturally and symbolically defined.

The mandate from the Norwegian Parliament was to restore the area “back to its original state” being an ambitious and inherently elusive goal for several reasons: (i) some landforms, once damaged, cannot be restored; (ii) even before the 1920s, the area was shaped by human and livestock activity, such as grazing; and (iii) the climatic conditions at the end of the 1920s—marking the close of the Little Ice Age—have since changed dramatically. These changes have deeply affected local ecosystems, glacier mass balance, runoff patterns, and permafrost. In fact, long-term ground temperature monitoring near Mt. Snøhetta has shown significant permafrost degradation and deepening of the active layer (Isaksen et al. 2011, 2022).

In 2018, the Snøhetta massif was incorporated into an expanded national park spanning 1830 km^2^. The park is classified by the International Union for Conservation of Nature (IUCN) as Category II, which allows for sustainable human activities within its boundaries (Dudley 2008; Dudley et al. 2013). One of the key factors enabling such a long-term, well-funded restoration effort is land ownership: the state owns the area and has the authority to fund and execute restoration programs. While Dovre serves as a model for mountain restoration, many such efforts will, however, confront far more complex legal and jurisdictional challenges that will require different approaches than those successfully implemented here.

Together, these cases highlight different pathways through which mountains become socially and politically significant: mythological (Parnassos), aesthetic and geological (Dolomites), and restorative (Snøhetta). While each case reflects European contexts, they illustrate broader tensions in defining mountains that arise wherever human engagement and valuation intersect with geomorphology. The subsequent discussion integrates these perspectives and considers their policy relevance.

## Discussion

In the preceding section, we outlined three different but complementary mountain perspectives from three European countries. These do not dwell on technical definitions of what constitutes a mountain, but rather on what shapes our perception of this particular landform and why we attribute value to it. We posit that this context is relevant for how definitions of mountains can be understood, challenged, and perhaps expanded.

### The Næss dilemma

A common denominator for Mt. Snøhetta, Mt. Parnassos, and the Dolomites is the seminal importance of human interaction with them. In all cases, humans have repeatedly engaged with these mountains for thousands of years, and their histories have become ingrained in national and international discourses. As a result, they and other similar ranges have likely influenced how Western culture understands what makes a mountain.

Given that nature is being diminished (e.g., Keck et al. 2025), and that regulations and socio-economic barriers limit mountain access, it is pertinent to ask how changing interactions affect both our understanding and definitions. Consequently, our entry to the following discussion centers around a set of pivoting points we have chosen to call “the Næss Dilemma,” after the philosopher Arne Næss (1912–2009), whose contribution to deep ecological thinking was connected to both his personal and professional relationship with mountains:

1. Unless we experience mountains, we will not develop empathy with them.
2. Unless we have empathy with mountains, we will not properly value them.
3. You cannot develop empathy with mountains unless you visit them, live in them, and/or work and play in them.
4. Human presence does not come without an impact (cost); our presence changes the mountains from what they were prior to our entry.
5. If we are to protect and restore mountains, we need to interact with them.

The Næss Dilemma does not require a formal definition, presupposing that most people recognize a mountain without one. The decisive factor is cultivating a relationship because only then can one ascribe value (Næss 2016).

This makes it very different from scientific, political, or administrative motivations for constructing formal, near-universal definitions. In those cases, values are often the premise from which needs arise whether for taxation, exploitation, or protection. We note that the debate about how to best define mountains occurs largely within the natural realm of science. Scientific approaches are often developed in response to administrative or political needs (Price et al. 2019). This approach also assumes that a “universal definition” can indeed be constructed which is a notion that is both controversial and far from trivial (Smith and Mark 2003).

### The ‘true value’ of a mountain

Mountains provide a set of ecosystem services: they release and bind carbon, and they impact—and are impacted by—weather and climate (Dahlen and Suppe 1988; Molnar and England 1990; Whipple et al. 1999). The strongest recognition of mountain value is arguably legal protection, if effectively enforced. There are different levels of protection, with the best-known label perhaps being a ‘national park’ although this means different things in different countries (Sax 1980). Moreover, such areas may also include privately owned pockets, often referred to as ‘inholdings’.

The term ‘national park’ reflects two ideas: ownership (national, regional, or private) and recreational space (‘park’ as controlled nature). Joseph L. Sax also pointed out that the early wave of American national parks “aligned neatly with nationalistic needs and the desire to equal European civilization” (Sax 1980). In the United States, national parks also serve as microcosms that embody longstanding conflicts between Indigenous peoples and political authorities (Spence 1999).

With judicial authority comes the power to shape nature according to certain ideals or simply to leave it alone. The late Mark Sagoff argued that the establishment of a national park allows people to “demand that the mountains be left as a symbol of the sublime, a quality which is extremely important in our cultural history, rather than be turned into an expression of the soft life, which is not” (Sagoff 1974, p. 266). In this context, efforts to restore nature may appear as a similar striving to secure the ‘sublime’ traditionally associated with national parks.

The large restoration project at Mt. Snøhetta in central Norway was initially aimed at returning the area to its ‘original state’ despite the fact that the physical conditions for that reference state no longer exist. The underlying idea seems to be that the ‘true value’ of nature equates with a condition unspoiled by humans—or more explicitly, a nature without humans—as though humans are not an integral part of nature.

The reference to ‘true value’ resurfaces in several works on nature and mountains, such as the European Environment Agency’s 2010 report Europe’s Ecological Back bone: Recognizing the True Value of Our Mountains. The title suggests both that mountains possess a ‘true value’ and that the recognition of this value depends on the choice of method. If one indeed can determine a true value for mountains—where some approaches equate this with a pristine or original state, while others (e.g., IPBES 2019) emphasize multiple, diverse values including cultural, spiritual, ecological, and material dimensions—then one can also estimate the cost of degradation and understand what may be irretrievably lost.

This line of reasoning typically hinges on an ‘objective definition’ of when a mountain is a mountain which is a formal necessity for any attempt to quantify the spatial distribution of mountains on this planet. Indirectly, this underscores the urgency of including the ‘missing mountains’, those partially or wholly embedded in ice or covered by seas and oceans (cf. Harris et al. 2014) as this would improve any attempt to describe Earth’s surface as an entity independent of what lies on top of it.

These ‘missing mountains’ challenge the Næss Dilemma in obvious ways: they are inaccessible to most people, making physical interaction impossible. They can, of course, be visualized using available data, enabling a form of ‘digital sensing’ (cf. Tress and Tress 2003). But the question remains: can this adequately substitute for the sensory experience of the landform that Næss referred to? As technology advances and the downwasting of ice sheets and glaciers continues, many hidden mountains will eventually become accessible, opening them first to scientific exploration followed by economic interests such as extractive industries, tourism, or bioprospecting. Safeguarding these and other mountains may well require a formal definition to facilitate protection, one that is easier to administer and less costly than future restoration.

### Nature as external to man, or nature as us?

Piccardi et al. (2008) notes that the true origin of the myth of the Oracle may lie in the dynamic movements of the mountainous landscape itself: earthquakes, avalanches, seismic ruptures, and bursts of fumes. For a thousand years, these powerful events were interpreted, performed, and retold by humans—time and time again. And somehow, throughout these persistent interpretations, humans separated themselves from their initiator as if curious to become the sole protagonists of world events. “Know thyself” was reduced to knowing the human self alone, even though a holistic understanding of nature persisted in many Indigenous communities.

In 2017, the Whanganui River in Aotearoa/New Zealand became the first river in the world to be granted legal status equivalent to that of a person with citizenship; a legal designation that, in some ways, surpasses national park status, particularly in terms of legal sanctions. This law followed a 150-year-long struggle by the Whanganui people. Their relationship with the river is encapsulated in the Māori saying, “Ko au te Awa, ko te Awa ko au” (“I am the River, the River is me”) (e.g., Macklin and Macklin 2021).

The idea that nature should be granted intrinsic value and protected through legal recognition was also championed by philosophers such as Arne Næss, although he avoided anthropomorphizing nature. For Næss, nature’s intrinsic value did not rest on its potential benefits to humans, but on its own participation in ecosystems. We are all part of nature: entangled within it and dependent on it. This aligns with Karl Polanyi’s argument in The Great Transformation, where nature has what Fred Block later referred to as “a sacred dimension” (Block 2021 p. 15).

Mountains are not only physical landforms but also governance challenges. The way they are defined has direct implications for policy, since statistical delineations underpin international biodiversity targets, protected area designations, and restoration priorities (e.g., Price et al. 2019). Our analysis suggests that narrow or universal definitions risk obscuring cultural diversity in how mountains are perceived and valued. By integrating scientific criteria with local narratives, policymakers can design protection and restoration strategies that are both scientifically robust and socially legitimate. This is particularly important given the rising attention to mountains in climate adaptation, biodiversity conservation, and Indigenous rights debates (e.g., Fernández-Llamazares et al. 2024). Mountains are recognized by the Intergovernmental Science-Policy Platform on Biodiversity and Ecosystem Services (IPBES) as critical biodiversity hotspots that support a substantial share of global ecosystem services and cultural values, yet remain highly exposed to climate impacts (IPBES 2019).

Recent IPBES assessments also highlight the need to integrate Indigenous and local knowledge systems into nature governance, underscoring that inclusive valuation frameworks are essential for sustainable and just conservation (IPBES 2022). Paraphrasing the geographer Denis Cosgrove (1945–2008) one can say that ‘mountains are a part of the earth surface that humans occupy, transform and occasionally aspire to transcend from. And that claim to truths about it is set by their context be that politics, culture or myths’ (Cosgrove 1985).

## Conclusion

Mountains have been critical for the evolution of humankind and its ensuing civilizations. The longstanding and life-giving relationship between mountains and humans has, throughout history, shaped our perception of what they are and how we relate to them. In early civilizations, supernatural beings such as gods made their homes in the mountains. There, they could be reached by those in search of answers about the future as was the case with the Oracle of Delphi on Mount Parnassos. In other words, nature, in the form of mountains, can provide insights to those who actively seek them.

How we interact with mountains has evolved. We have found ways to protect mismanaged mountains from human activity by, for instance, placing them under patronage as with the Dolomites in Italy, which in 2008 were added to the UNESCO World Heritage list. Yet mountains are subject to persistent climate change and continued human pressure. The combined effects of these forces are altering how we value mountains compelling us to create new ways of limiting negative impacts.

One established solution has been to place mountains under legal protection by designating them as national parks. This level of protection still grants some access, but it does little to counter the broader impacts of climate change. The most extreme measure available is to restore mountains to a ‘former state’, as in the case of Snøhetta Mountain. The main motivation for this approach appears to be a modern desire for unspoiled nature, for recreation, for self-discovery, and possibly for the growing realization that rich biodiversity can enhance resilience to external pressures like climate change.

We have explored how various definitions of mountains come with limitations and advantages. Most quantitative methods do not account for large portions of Earth’s surface when producing ‘authoritative statistics’—particularly mountains embedded in ice sheets. Future estimates of mountains beneath oceans and seas will add to the growing pool of definitions. We anticipate that such mapping will better include these ‘hidden mountains’ in global statistics, though whether that will lead to stronger protection remains to be seen.

The Sisyphean effort of producing a universal definition of what a mountain is will surely persist as will the many ways in which humans interact with, perceive, and (de)value mountains. The “Næss Dilemma,” which suggests that we cannot develop empathy for mountains without interacting with them, reminds us that protecting them from climate change and human pressure will remain a challenge regardless of one’s ideological footing.
